# Nanomaterials to Enhance Food Quality, Safety, and Health Impact

**DOI:** 10.3390/nano10050941

**Published:** 2020-05-14

**Authors:** Sergio Torres-Giner, Cristina Prieto, Jose M. Lagaron

**Affiliations:** Novel Materials and Nanotechnology Group, Institute of Agrochemistry and Food Technology (IATA), Spanish Council for Scientific Research (CSIC), Calle Catedrático Agustín Escardino Benlloch 7, 46980 Paterna, Spain; cprieto@iata.csic.es

**Keywords:** antioxidants, oxygen scavengers, antimicrobials, bioactives, barrier, active packaging, controlled release, migration, nanoencapsulation, nanocomposites, Circular Bioeconomy

## Abstract

Food quality and safety are key aspects to guarantee that foods reach consumers in optimal conditions from the point of view of freshness and microbiology. Nanotechnology offers significant potential to secure or even enhance these aspects. Novel technologies, such as nanofabrication and nanoencapsulation, can provide new added value solutions for the fortification of foods with bioactives and targeted controlled release in the gut. Nanomaterials can also support food preservation aspects by being added directly into a food matrix or into food contact materials such as packaging. Thus, nanomaterials can be leveraged in the form of nanocomposites in food packaging design by melt compounding, solvent casting, lamination or electrohydrodynamic processing (EHDP) to promote passive, active, and even bioactive properties such as barrier, antimicrobial, antioxidant, and oxygen scavenging roles and the controlled release of functional ingredients. These attributes can be exerted either by the intended or non-intended migration of the nanomaterials or by the active substances they may carry. Lastly, nanomaterials can be advantageously applied to provide unique opportunities in Circular Bioeconomy strategies in relation to the valorization of, for instance, agro-industrial wastes and food processing by-products.

The use of nanotechnology for food applications is a rapidly evolving field, and given the specific properties of nanomaterials and their tremendous potential in food science and packaging technology, an increased number of innovations that contribute to improving food quality, safety, and health impacts are foreseen. This Special Issue compiles twelve articles and three reviews written by researchers and technologists that, together, constitute an interesting and multi-disciplinary approach to addressing all these topics.

In food packaging applications, nanocomposites represent an alternative to conventional technologies for improving biopolymer passive properties by adding nanoparticles for which at least one dimension is in the nanometer range. Cellulose is an appropriate candidate to be used as a reinforcing nanomaterial since it is a fibrous, tough, and water-insoluble material, also being the most abundant renewable biopolymer produced in nature. However, cellulose can be obtained not only from vegetables (e.g., plants and some alga species) but also from microbes (e.g., bacteria). The so-called bacterial cellulose (BC) is constituted of fermented fibers that, in contrast to cellulose plant fibers, consist of pure cellulose, so they display higher crystallinity and show improved properties such as high purity (with the absence of lignin and hemicellulose), ultrafine fibrous structure, low density, high water-retention capacity, and biocompatibility. Wardhono et al. [1] developed a fast, highly-efficient, and eco-friendly preparation method for the extraction of cellulose nanocrystals (CNCs) from BC. This method consisted of a two-step process, namely the partial depolymerization of BC under ultrasonic irradiation and the extraction of crystalline regions using microwaves, assisted by manganese (II) chloride (MnCl_2_)-catalyzed hydrolysis, successfully yielding bacterial cellulose nanocrystals (BC-NCs) with similar features to commercially available nanocrystalline cellulose. In another study, Guzman-Puyol et al. [2] prepared all-cellulose nanocomposites with potential applications in compostable food packaging by using a simple method consisting of dissolving cellulose in a trifluoroacetic acid/trifluoroacetic anhydride 2:1 (vol/vol) mixture and subsequently adding different cellulose nanofibers (CNFs) dispersed in chloroform. The best performance was achieved for concentrations of nanofibers ranging from 5 to 9 wt%, maintaining excellent transparency, improving the mechanical properties, and reducing the water permeability. Nevertheless, more insights into the life cycle risk assessment and environmental health and safety roadmap of the potential risks from the inhalation of cellulose nanomaterials are needed to advance the safe commercialization of these materials. To this end, Ede et al. [3] summarized, in a review, the currently available published literature regarding the cellulose nanomaterial inhalation hazard and evaluated the quality of the studies for risk assessment purposes. It was concluded that short-term exposure to cellulose nanomaterials could result in transient inflammation, similarly to other poorly soluble and low toxicity dusts. However, several data gaps still remain, and there is a lack of understanding of the effects of long-term and low-dose exposures that represent realistic workplace conditions, essential for a quantitative assessment of the potential health risks.

Many efforts made to prevent food deterioration and improve the effectiveness of active compounds have triggered recent innovations in the field of food packaging. In this context, the passive role of traditional packaging in the protection and marketing of a food product has evolved into a novel “extra” function as a carrier of active compounds. Active packaging technology is based on the intentional incorporation of active agents into a packaging material that would then be released into and/or absorb substances from the packaged food or the environment surrounding the food. Concerning active packaging materials, these can be classified as either active-scavenging types (e.g., oxygen scavengers) or active-releasing types (e.g., antioxidants and antimicrobials). The development of novel antioxidant packaging using nanotechnology provides an opportunity to extend the freshness of food products by absorbing the compounds that deteriorate the food such as oxygen or free radicals. In a research article, Vera et al. [4] demonstrated that active packaging bags based on selenium nanoparticles (SeNPs) between 50 and 70 nm can prevent the oxidation and extend the shelf life of hazelnuts, walnuts, and potato chips. The metalloid nanoparticles were incorporated in solution into the adhesive layer of a polyethylene terephthalate (PET)/adhesive/low-density polyethylene (LDPE) multilayer, performing as a strong free radical scavenger. The newly developed active packaging material was also tested in food industrial lines where cooked ham and chicken as well as a ready-to-eat vegetable mixture seasoned with butter were industrially packaged with the antioxidant multilayer, and improvements higher than 25% were obtained. This study thus opened the door to new developments of active materials containing nanoparticles in which the nanomaterials are not in direct contact with the food but act to protect the food via the packaging, reducing the formation of lipid off-flavors that cause food product rejection by consumers. In the field of active-scavenging packaging, also, Cherpinski et al. [5] developed oxygen scavenging films consisting of poly(3-hydroxybutyrate) (PHB) containing palladium nanoparticles (PdNPs) by electrospinning and annealing treatment at 160 °C. The PdNPs were pretreated with surfactants permitted for food contact applications to optimize their dispersion and distribution in PHB. The PHB/PdNP nanocomposite films had high oxygen scavenging performance, although the activity was reduced after annealing. In a second study [6], water barrier and oxygen scavenging multilayer films were developed by the coating of paper with electrospun PdNP-containing PHB and poly(ε-caprolactone) (PCL) fiber mats followed by the annealing of the whole bilayer. The PdNPs-containing PCL/paper multilayer exhibited higher oxygen scavenging capacity than the homologous based on PHB due to the higher oxygen permeability of PCL and the higher dispersion of PdNPs. The active multilayered biopapers developed are of significant relevance to the development of the next generation of fully biodegradable barrier papers of interest in food packaging.

Active-releasing type packaging can additionally provide novel functions, such as aromatic, antioxidant, and long-term antimicrobial properties. In this regard, the electrohydrodynamic processing (EHDP), including both electrospinning and electrospraying techniques, supposes an innovative nanofabrication approach for the development of active food packaging coating and interlayer materials. This technology employs a high-voltage electric field imposed on a polymer solution to create polymer fibers or beads with diameters ranging from below 100 nm to several micrometers. As an example of this, Arrieta et al. [7] developed bio-based and biodegradable bilayer systems with antioxidant properties. The outer layer was based on a compression-molded poly(3-hydroxybutyrate-*co*-3-hydroxyvalerate) (PHBV) film, while the inner active layer was formed by electrospun nanofibers based on polylactide (PLA) and PHB blends (3:1, wt/wt) loaded with 1 and 3 wt% of catechin, a natural flavonoid with antioxidant activity. Moreover, in order to increase the stretchability and to facilitate the electrospinning process, 15 wt% of oligomer of lactic acid (OLA) was added as a plasticizer. The obtained bilayers showed effective capacities to release antioxidants into a fatty food simulant, while the bilayer films showed appropriate disintegration under compost conditions in less than three months (90% of disintegration according to ISO 20200), thus showing their potential as compostable active packaging for fatty food products. In other work, Salević et al. [8] prepared novel active films made of PCL containing a solid dispersion of sage (*Salvia officinalis* L.) extract at loading contents of 5, 10, and 20 wt% by means of the electrospinning technique and subsequent annealing. The thermal post-treatment yielded thin and hydrophobic films with good contact transparency, showing high free radical scavenging ability and also strong activity against foodborne pathogens. The evaluated characteristics of the PCL-based films containing sage extract suggest great potential in active food packaging applications with the aim of preventing oxidation processes and microbiological contamination or growth.

As shown above, the design of active-releasing type packaging materials represents a very dynamic field, and multiple functions can be added by electrospinning. For instance, active packaging systems can not only extend the shelf life of food products and reduce food waste by maintaining the quality of food products for longer but also increase product safety by securing the foods against pathogens. In this regard, antimicrobial packaging applications can improve food microbial safety by preventing the growth of spoilage and/or pathogenic microorganisms. In this area, Melendez-Rodriguez et al. [9] developed electrospun PHBV films with long-term antimicrobial capacities by incorporating mesoporous silica nanoparticles in the 2.5–20 wt% range. The mineral nanoparticles containing 50 wt% of eugenol were loaded into PHBV by electrospinning, and the resultant electrospun composite fibers were annealed at 155 °C to produce continuous films. The electrospun PHBV films with loadings above 10 wt% of mesoporous silica nanoparticles containing eugenol successfully inhibited bacterial growth, and their antimicrobial activity increased after 15 days when stored in hermetically closed systems due to the volatile portion accumulated in the system’s headspace and the sustained release capacity of the active films. The resultant biopolymer films were reported candidates to be applied in active food packaging applications to provide shelf life extension and food safety. Similarly, Figueroa-Lopez et al. [10] encapsulated oregano essential oil (OEO), rosemary extract (RE), and green tea extract (GTE) in ultrathin fibers of PHBV derived from fruit waste using solution electrospinning, and the resultant electrospun mats were annealed to produce continuous films. The incorporation of the natural extracts and essential oils resulted in PHBV films with a relatively high contact transparency and hydrophobicity, of which the electrospun OEO-containing PHBV films presented the highest antimicrobial activity against two strains of food-borne bacteria. Furthermore, these active films also showed the most significant antioxidant performance, which was ascribed to the films’ high contents of carvacrol and thymol. One can expect that these new materials can be applied as new active coating or interlayer systems in the design of active food packaging structures in the frame of Circular Bioeconomy to prolong the shelf life of foods and delay the proliferation of microorganisms and enzymatic oxidation of foodstuffs.

Nanotechnology also opens up new opportunities in the field of intelligent or smart packaging. This concept relates to future technologies that allow packaging to contain, evaluate, and transmit relevant information. For instance, the use of electrically conductive polymer-based materials can serve to create “smart” labels or tags that can enable the tracking and monitoring of the conditions and quality of the packaged products, such as food freshness, from the production line to the end user. In our recent study [11], graphene nanoplatelets (GNPs) were embedded in poly(ethylene-*co*-vinyl alcohol) (EVOH) nanofibers by electrospinning to create electrically conductive thin layers with nanofiller contents of only 0.5 wt%. The electrospun mats were also thermally post-treated at 158 °C to produce continuous and contact transparent EVOH films to be applied as smart tags in film interlayers. In recent years, the fast development of various nanomaterials has also opened up new possibilities for biosensing signal amplification. Among them, Wang et al. [12] demonstrated the potential of protein-inorganic hybrid nanoflowers to maintain or even increase the activity of the proteins and effectively amplify the detection signals. A new enzyme-free assay was explored to detect *S. typhimurium*, being able to detect bacterial cells at levels as low as 28 CFU/mL within two hours. One of the most interesting and promising nanomaterials for the detection of various species (e.g., bacteria, cells, nucleic acids, molecules, ions, etc.) are colloidal semiconducting quantum dots (QDs). Lesiak et al. [13] reviewed the use of QDs to enable high-throughput and mobile diagnostic platforms for screening for pathogens and toxins immediately in the field. Nevertheless, it was concluded that a universal sensor for different types of food samples is currently a challenge because of the inherent complexity of biological samples.

As nanotechnology provides “a new dimension” accompanied by novel or modified properties conferred to many current materials, it can also be successfully used for the production of a new generation of nanoformulated human dietary supplements, functional foods, or nutraceuticals. A bioactive or functional ingredient is any food or food component providing health benefits beyond basic nutrition and reducing the risk of disease. Nutraceuticals are bioactive substances or a mixture of bioactive compounds based on food, herbal, or other natural products that are used in the form of pharmaceutical formulations such as tablets, capsules, drops, or liquids. In contrast to drugs, in all these cases, the bioactives are present in low concentrations. The intake of nutraceuticals and fortification of edible products with bioactive components have both become increasingly popular in modern society, since they can help to balance the total nutrient profile of a diet, supplement nutrients lost in processing, and, thus, correct or prevent insufficient nutrient intake and associated deficiencies. In this context, the review performed by Jampilek et al. [14] compiles the current state-of-the-art in the development of bio-based nanoscaled delivery systems such as nanocapsules, nanofibers, or nanoparticles, among others, to stabilize and enhance nutraceuticals’ functionality in various food products and drinks during food processing or digestion. In the field of the nanoencapsulation of bioactives, the electrospraying process has shown to be highly suitable for entrapping thermolabile compounds due to its high efficiency and the fact that it can be performed under room conditions. In this regard, Ramos-Hernández et al. [15] studied the ability of high degree of polymerization Agave fructans (HDPAF) to form capsules by electrospraying and assessed the viability of this polysaccharide as a nanoencapsulating material for β-carotene by direct electrospraying and by electrospraying coating (EC). The findings showed that the EC method, based on a three-step process, yielded ultrathin particles with higher bioactive/polysaccharide ratios. Moreover, the HDPAF nanoparticles obtained by the EC method showed good photoprotection and improved the stability of β-carotene, being promising for human consumption.

In summary, the Special Issue entitled *Nanomaterials to Enhance Food Quality, Safety, and Health Impact* in *Nanomaterials* reflects the high diversity and creativity of new nanomaterials and nanofabrication processes that are rapidly developing in the research fields of food science and packaging technology. This focus will contribute to the research interest in the field of food quality and safety, providing our readership with a multi-faceted scenario that outlines the importance of food-related nanotechnologies and their wide-ranging applications. It is also expected that the present Special Issue will encourage new multidisciplinary research on nanomaterials to promote health benefits, broadening the range of potential practical uses. However, all nanomaterials applied in the food industry should be used advisedly and only after the in-depth investigation of cytotoxicity due to possible increased toxicity effects induced by their high surface reactivity. In food packaging, future research efforts will, particularly, have to be coupled to new studies devoted to the risk factors associated with the potential migration of nanoparticles from the packages and their impact on both human health and the environment in the long term.
